# Development and validation of the physical exercise program “Active Mums” for postpartum recovery: application of the CReDECI-2 guidelines

**DOI:** 10.1186/s12884-024-06387-1

**Published:** 2024-05-20

**Authors:** Carla Brites-Lagos, Cristiana Maranhão, Anna Szumilewicz, Rita Santos-Rocha

**Affiliations:** 1 ESDRM Sport Sciences School of Rio Maior – Santarem Polytechnic University, Rio Maior, Portugal; 2 Gdansk University of Physical Education and Sport, Gdansk, Poland; 3 SPRINT – Sport Physical Activity and Health Research and Innovation Center – Santarem Polytechnic University, Rio Maior, Portugal

**Keywords:** Postpartum, Physical activity, Exercise, Complex intervention

## Abstract

Postpartum physical activity is a public health issue. Reporting on the quality of exercise interventions designs must be ensured in view of the reproducibility and successful implementation of such studies. The objective was to develop and preliminary validate a physical exercise program for postpartum recovery, aiming to promote physical fitness and health of the new mothers. The study was carried out through the three stages of development, piloting, and evaluation. The Consensus on Exercise Reporting Template (CERT) was used to describe the postpartum exercise program. The Criteria for Reporting the Development and Evaluation of Complex Interventions in Healthcare (CReDECI2) was followed to develop and preliminary validate the program. A tailored postpartum exercise program was developed based on evidence-based international recommendations to be implemented by qualified exercise professionals. A pilot intervention of 16 weeks was carried out, engaging a group of postpartum women. The viability of the program was subsequently evaluated by all participants. The present work provided guidance to develop a study protocol with a larger sample in order to prove the effectiveness of a supervised postpartum exercise program on selected parameters of health.

## Background

Regarding the effects of physical activity on maternal health during pregnancy and postpartum, the public health importance of increasing physical activity in women of childbearing age before, during, and after pregnancy is substantial [1]. In 2020, the World Health Organization (WHO) issued updated physical activity and sedentary behavior guidelines for various populations, reinforcing the need for national policies to include and monitor these subpopulations [2]. For the first time, specific advice for women during pregnancy and the postpartum period was included [2], and therefore, it supports the importance of monitoring and evaluating the implementation and impact of specific national guidelines on physical activity and exercise during these periods of life.

Exercise is a simple, low-cost intervention that can play an important role in health and well-being during the postpartum period [3]. Specific programs for this stage of life can increase physical activity and consequently, potentially improve women’s health and fitness, as shown in the umbrella review by Dipietro et al. [1]. Since 2019, updated international guidelines for exercise during pregnancy and postpartum from Canada [4], USA [5], Brazil [6], and Australia [7] have been published. However, these guidelines lack specific postpartum contents, and only one consensus paper supported by the International Olympic Committee (IOC) addresses exercise and postpartum in recreational and elite athletes [8]. Clear guidance and reporting of exercise interventions is critical for interpreting and translating research into clinical practice. Nevertheless, the available official guidelines lack structured methodologies to design and implement effective and safe exercise programs [9, 10]. Therefore, it is important to know more precise information about the type of exercise, intensity and frequency, in order to fully understand the intervention and how to replicate it.

The Consensus on Exercise Reporting Template (CERT) by Slade et al. [11] is a tool used to describe the components of interventions based on exercise programs. The Criteria for Reporting the Development and Evaluation of Complex Interventions in healthcare (CReDECI2) by Möhler et al. [12], allows describing all the relevant methodological aspects related to the process of research and development, piloting and evaluation of a complex intervention, which can be applied to exercise programs.

Therefore, the objective of this study was to develop and preliminary validate a physical exercise program “ACTIVE MUMS”, specific for postpartum recovery that aims to promote maternal health and physical fitness, helping women to either commence or resume an active and healthy lifestyle after giving birth.

## Methods

### Study Design

The Criteria for Reporting the Development and Evaluation of Complex Interventions in healthcare (CReDECI2) was applied in the development and validation process of an exercise program for postpartum recovery as a complex intervention.

## Participants

Seventeen participants were involved in the validation process, six specialists in physical exercise with a doctorate or master’s degree in exercise/sports science and 11 women who were in the postpartum period, who participated in the pilot intervention.

## Instruments

The Consensus on Exercise Reporting Template (CERT) was followed to describe the postpartum exercise program [11], and the Criteria for Reporting the Development and Evaluation of Complex Interventions in healthcare (CReDECI 2) was followed to develop and preliminary validate it [12].

## Procedures regarding the development of the postpartum exercise program

The exercise program was designed through three stages proposed by the CReDECI2 guidelines: development, piloting and evaluation, and described in alignment with the CERT. The postpartum physical exercise program was developed by exercise specialists. The periodized training plan includes a variety of exercises to promote cardiorespiratory fitness, posture, strength, flexibility, balance, abdominal recovery, and pelvic floor muscle training.

## Procedures regarding the piloting and evaluation of the postpartum exercise program

The dissemination process, recruitment of the target population and implementation of the pilot study in a training studio were planned. An in-person or online pilot intervention of 48 sessions over 16 weeks was developed between October 2021 and June 2022, in Leiria, Portugal. The pilot exercise intervention was delivered by an exercise physiologist. All women in the postpartum period (4–12 weeks) received a medical consultation by a gynecologist before being included in the pilot intervention. The inclusion criteria were: women in the (4–12 weeks) postpartum period, aged between 18 and 45 years old, without medical contraindications for the practice of physical exercise, according to ACOG [5], primigravida or multigravida women. Exclusion criteria were: any medical contraindications for physical exercise.

Participants were informed about the objectives and characteristics of the intervention and an informed consent was obtained. Eleven women with uncomplicated pregnancies and deliveries participated in the pilot intervention, after single pregnancies, between four and 12 weeks postpartum, without complications or contraindication to exercise, mean age of 31 years. Before the exercise intervention, five women had a body mass index (BMI) above the healthy limit, i.e., between 25.0 and 29.9 kg/m^2^, according to Jensen et al. [13]. No adverse events were reported during the pilot intervention.

## Ethical considerations

Healthy women in the postpartum period were invited to participate in the pilot intervention, free of charge. An informed consent was signed prior to participation and women were informed about the objectives, the nature of the study, the potential benefits, participation requirements and their right to withdraw from the study at any time, without any consequences. All exercise sessions were conducted by a qualified exercise physiologist. All clinical appointments were conducted by a gynecologist. The study was conducted in accordance with the Helsinki Declaration [14]. This study is part of the study protocol that has been approved by the Ethics Committee of the Polytechnic Institute of Santarém, Portugal (approval number 9-2021-ESDRM).

## Results

### First stage: development

Item 1 – Description of the intervention’s underlying theoretical basis.

Physical activity during the postpartum period is a public health issue [1]. The postpartum is a period marked by profound changes in women at the physical, psychological and physiological level. However, usually, postpartum women are not encouraged to practice exercise, except for strength training of the pelvic floor muscles. Therefore, they are likely to develop complications such as pelvic pain, lumbar pain, abdominal diastasis, and psychological complications, such as postpartum depression [8]. Almost half of normal weight women and 2/3 of overweight/obese women exceed the Institute of Medicine (IoM) gestational weight gain guidelines for pregnancy [15], with this weight retention being associated with increased risk of obesity, cardiovascular disease, and type II diabetes during middle age [8].

The benefits of exercise in the postpartum period include promoting the return to pre-gestational weight, decreasing the risk of developing future chronic health conditions, and improving physical fitness [1]. In addition to these benefits, several studies have shown that exercise in postpartum is effective in reducing symptoms of depression [16, 17], musculoskeletal disorders [18, 19], and fatigue [20]. In line with evidence-based research, exercising at this stage proves to be extremely important, making it important to understand and implement effective strategies and programs that promote it.

Specific guidelines for physical activity during the postpartum period are embedded in the documents supported by the American College of Obstetricians and Gynecologists (ACOG) [5], the Brazilian Society of Cardiology (SBC) [6], and Sports Medicine Australia (SMA) [7]. One consensus paper supported by the International Olympic Committee (IOC) addresses exercise and postpartum in recreational and elite athletes [8]. The U.S. Department of Health and Human Services (USDHHS) [21], the UK Chief Medical Officers [22], the WHO [2], and the American College of Sports Medicine (ACSM) [23] issued guidelines on physical activity and sedentary behavior for postpartum women, reinforcing the need for national policies to include and monitor this subpopulation. International entities such as the ACOG [24], the UK Chief Medical Officers [25], the National Health Services, UK [26], and the Department of Health – Victoria State Government, Australia [27] also published practical recommendations for health and physical activity during the postpartum period, in simple and accessible language. Santos-Rocha and Szumilewicz [10] published an updated textbook chapter about “Exercise Prescription and Adaptations in Early Postpartum” including specific and practical contents. These updated practical recommendations helped to guide the development of the proposed postpartum exercise intervention.

Item 2 – Description of all intervention components, including the reasons for their selection as well as their aims/essential functions.

The postpartum exercise program was described using the 16 CERT items [11] as follows.

Item 2.1 - Type of exercise equipment.

The tailored postpartum exercise program required equipment such as mattresses, Swiss balls™, elastic bands (from light to medium resistance levels), free weights (dumbbells from 0.5 to 3 kg), kettlebells (12 to 16 kg), TRX™, softballs, ergometers, and pulleys. The virtual training program did not include ergometers and pulleys.

Item 2.2 - Qualifications, expertise and/or training of the exercise professionals.

The postpartum exercise program was delivered by exercise physiologists, holding a bachelor’s degree in Exercise Sciences / Sport Sciences / Physical Education and a master’s degree in Physical Activity and Health, and with expertise and experience with pregnancy and postpartum exercise. These exercise professionals are key elements of structured training for participants in the postpartum, and with knowledge and skills to report and refer to health professionals, if necessary.

Item 2.3 - Description whether exercises are performed individually or in a group.

The postpartum exercise program consisted of a personalized training program carried out in a small group or individually. The groups, in-person or online, had up to four participants. It was possible to adapt the structure of the group sessions to online sessions, due to the confinement of some participants, caused by the COVID-19 pandemic, and due to participants preferences.

Item 2.4 - Description whether exercises are supervised or unsupervised; how they are delivered.

The postpartum exercise program was a supervised training program provided in-person or online by an exercise professional whose qualifications are described in item 2.2. Supervision is needed to monitor performance and adherence, provide feedback on proper technique, adapt exercises, if necessary, ensure safety, and refer to a healthcare professional if necessary. Lack of monitoring and loss to follow-up were higher and adherence to intervention was lower for unsupervised versus supervised studies [28]. In parallel to the program, participants were advised to be physically active.

Item 2.5 - How adherence to exercise is measured and reported.

The exercise professional checked participation in each of the 3 available exercise sessions per week. The frequency rate in the postpartum exercise program was calculated by dividing the number of sessions attended by the number of sessions scheduled.

Item 2.6 - Motivation strategies.

Motivation strategies were applied through direct interaction between the exercise professional and the participants. Extensive advice and clarification on the importance of regular and consistent exercise was provided through clear instructions.

Private groups were also created on social networks (WhatsApp, Facebook and Instagram) to allow sharing of information and social interaction.

Regarding health parameters, physical activity and quality of life, participants were instructed to complete the following questionnaires before, during and after the exercise program, in order to check progress with regards to goals and potential contraindications for exercising:

- PAR-Q+ Physical Activity Readiness Questionnaire for Everyone [29], adapted to Portuguese [30].
- International Physical Activity Questionnaire (IPAQ) [31], adapted to Portuguese [32].
- World Health Organization Quality of Life (WHOQOL-Bref) [33], adapted to Portuguese [34].
- Pelvic Girdle Questionnaire (PGQ) [35], adapted to Portuguese [36].
- Roland-Morris Disability Questionnaire (RMDQ) [37], adapted to Portuguese [38].
- Fatigue Assessment Scale (FAS) [39], adapted to Portuguese [40].
- Edinburgh Postpartum Depression Scale (EPDS) [41], adapted to Portuguese [42].

These tools were chosen because they may comprehensively evaluate outpatient postpartum recovery. However, postpartum recovery is an underexplored area of obstetrics, and there is currently no consensus regarding which patient-reported outcome measure clinicians and researchers should use to evaluate postpartum recovery [43].

In addition to the questionnaires, functional assessment can be performed from the early postpartum [44]. Moreover, in a later stage, the assessment of the physical fitness components (body composition, posture, functional, cardiorespiratory, muscular resistance, flexibility) may motivate the participants to practice exercise, in order to monitor their progress and consequently obtain better results [23].

Item 2.7a - Decision rule(s) for determining exercise progression.

The exercise program was periodized in 3 weekly sessions of 60 min, referring to 3 mesocycles: adaptation (two weeks), improvement (six to eight weeks) and maintenance (four to six weeks). Each session followed the conventional structure proposed by ACSM [23] and addressed the components of health-related fitness. Postpartum exercise was planned according to the recommendations for physical activity of the main international organizations, already mentioned [5–8], and described by Santos-Rocha and Szumilewicz [10].

The intensity progression was determined through the Borg scale of perceived exertion and the talk test [23]. The goal is to achieve a light to moderate training level of perceived exertion (RPE) Borg scale (11–13 out of 20), which corresponds to 50% of the estimated maximum oxygen uptake and 60% of the estimated Maximum Heart Rate for age (HRmax) using the equation by Gellish et al. [45], at an effort level of RPE (14–15 out of 20) that corresponds to 75% of the estimated maximum oxygen uptake and 80% of the HRmax.

Regarding resistance training, the exercise physiologist adjusted the exercise intensity (load) as determined by the participant’s ability to complete 2–4 sets of 8–12 repetitions for a given exercise (0–60% of one repetition maximum for lower limb exercises) [23]. If exercise resulted in pain, discomfort or fatigue, the intensity would be reduced. In addition, participants received feedback on exercise progression.

Item 2.7b - How the exercise program progresses.

The progression of the exercise program was performed by increasing the complexity of the exercise (instability, reducing the number of supports), increasing the number of sets and/or repetitions, among other parameters such as cadence, rest periods, or weight, i.e., the external load, as well as the intensity (internal load). It also considered the discomforts of the woman and the improvement of her abilities.

Item 2.8 - Description of each exercise to enable replication.

The postpartum exercise program is described in the Manual “Prescription of Physical Exercise in the Postpartum Period” [46] published, so far, in the Portuguese language. In this manual, details are provided on the organization of the sessions and description of each exercise, considering equipment, position, adaptations, technique, number of repetitions and sets and safety considerations. Each exercise is illustrated by an image of each phase or form of performance to allow the replication. The manual is supported by freely accessible videos posted on the YouTube channel “Gravidez Ativa – Active Pregnancy”^1^.

Item 2.9 - Description of any home program component.

This program did not include a prescribed unsupervised home exercise. However, participants were encouraged to regularly perform stretching, breathing exercises and pelvic floor muscle exercises. It also encouraged the adoption of more active and healthy lifestyles, encouraging outdoor walks, as well as the adoption of healthy eating habits. This component was also supported by freely accessible videos posted on the YouTube channel “Gravidez Ativa – Active Pregnancy”^1^.

Item 2.10 - Description whether there are any non-exercise components.

The postpartum exercise-based intervention was supplemented by several psycho-social support strategies, such as educational information and lifestyle advice. Therefore, the non-exercise component of the program referred to the advice on the benefits of a healthy lifestyle (nutrition, sleep, etc.), physical activity in general and postpartum exercise in particular. This component was supported by the dissemination of the free downloadable guides “Active Pregnancy Guide - Physical activity, nutrition, and sleep” [47] (also available in English [48]), and “Promotion of physical activity and exercise during pregnancy and postpartum. Health professionals guide” [49] (also available in English [50]), by the organization of lectures, and by free access videos posted on the YouTube channel “Gravidez Ativa – Active Pregnancy”^1^.

Item 2.11 - Type and number of adverse events that occur during exercise.

No adverse effects occurred during the pilot intervention.

Item 2.12 - Setting in which the exercises are performed.

The in-person exercise sessions were delivered at the gym. The pilot intervention took place at *Lateral Performance* training studio, located in Gândara dos Olivais, Marrazes, 1.5 km from the center of Leiria, Portugal. It featured safety and hygiene conditions and equipment for small groups. The floor was not slippery and it was suitable for shock absorption. It was possible for the exercise professional to maintain eye contact with the group throughout the session and provide individual feedback. The training music was optional, having the equipment for this purpose.

As for the online training, the participants were advised of the care to be taken with the type of floor and the way in which they should be framed with the camera, to guarantee supervision.

Item 2.13 - Detailed description of the exercise intervention.

A typical 60-minute session was structured as follows (Table 1):

**Table 1:** – Structure of a typical 60-minutes session of the postpartum exercise program

Type of exercise	Description	Duration (min)	Duration (%)
Warm-up	The warm-up consists of a few minutes of an activity that will elevate heart rate followed by dynamic movements.	5–10 min	8–17%
Low-impact cardiorespiratory training	Includes aerobics, step or dancing exercises, or indoor cycling, or treadmill walking.	10–20 min	17–33%
Neuromotor training	Includes postural, balance and coordination exercises (e.g., barre, ball).	5–10 min	8–17%
Resistance training	Includes low to moderate resistance exercises for the core, lower and upper limbs, and back, performed with body weight, free weights, small equipment or machines.	20–25 min	33–42%
Pelvic floor muscle training (PFMT)	PFMT consists in the repetition of one or more sets of voluntary contractions of the pelvic muscles.	5 min	8%
Stretching	Includes exercises that increase the range of motion of joints, to improve blood flow through the body, balance, or flexibility.	5 min	8%
Breathing and relaxation	Includes breathing and relaxation exercises, usually in the lying position.	5 min	8%

*Note* further description of the type of exercises and its benefits can be found in ACSM [23]; further description of the postpartum exercise program can be found in Santos-Rocha and Szumilewicz [10] and Santos-Rocha et al. [46] (ebook in Portuguese); examples of workouts can be found on the YouTube channel “Gravidez Ativa – Active Pregnancy”^1^

A typical periodization was structured as follows (Table 2):

**Table 2:** – Periodization of the postpartum exercise program

Early postpartum (0–6 weeks)			Late postpartum
1–2 weeks	3–4 weeks	5–6 weeks	6/8 weeks+
Walking 10–30’ (daily)	Walking 10–40’ (daily)	Walking 10–60’ (daily)	Walking / Aerobics / Cycling / Swimming / Running 20–60’ (daily)
15–30’ Posture Stretching	20–35’ Posture Core Stretching	25–40’ Posture Core Functional Stretching	25–40’ Posture / Resistance Balance / Coordination Stretching / Flexibility
PFMT (daily)	PFMT (daily)	PFMT (daily)	PFMT (daily)

Item 2.14a - Description whether the exercises are generic or tailored.

The exercises were personalized, considering the needs and specificities of each woman.

Item 2.14b - Detailed description of how the exercises are tailored to the individual.

Each exercise included variations to increase or decrease intensity or complexity, depending on each participant’s skills and fitness level.

Item 2.15 - Decision rule for determining the starting level.

The pre-exercise assessment in the postpartum period would be compartmentalized into different parameters, such as: health, level of physical activity, quality of life, low back pain and pelvic pain, fatigue, depression and functional and fitness level. The exercise professional should be responsible for implementing the pre-exercise assessment procedures, interpreting the results of this screening, prescribing an appropriate starting level for each participant, as well as providing instructions to participants on which level to choose. Regarding these purposes, the following instruments could be used:

- Health: PAR-Q+ [29].
- Level of Physical Activity: IPAQ [31].
- Quality of life: WHOQOL-Bref [33].
- Pelvic pain: PGQ [35].
- Low back pain: RMDQ [37].
- Fatigue: FAS [39].
- Depression: EPDS [41].
- Fitness Level:
- Blood pressure, as well as resting heart rate and reserve and maximum heart rate calculated [23].
- Body composition: BMI; Waist-hip ratio; Electrical Bioimpedance [23].
- Postural Assessment: Static observation of the anatomical references, in the frontal and sagittal planes, verifying the symmetry in relation to the imaginary midline [44].
- Functional Assessment.

- DNS: The seated diaphragm test, Intra-abdominal pressure test, Quadruped rockforward test [51].
- FMS: Shoulder Mobility, Active straight leg raise, Deep Squat, Rotary Stability [52, 53].

- Cardiorespiratory Fitness: Rockport One-Mile Fitness Walking Test [54, 55].
- Muscular resistance: maximum number of arm extensions (push-ups) [23]; “Chair Stand Test” [56].
- Flexibility: V sit and reach test [23, 57].

Item 2.16a - Description how adherence or fidelity is assessed/measured: described in item 2.5.

Item 2.16b - Description of the extent to which the intervention was delivered as planned.

The pilot postpartum exercise program was delivered as planned and feasible in the prophylactic isolation of some participants, due to the online option. Most participants attended more than 90% of sessions. There were no dropouts.

Item 3 – Illustration of any intended interaction between different components.

During the pilot intervention, the participants gave their oral feedback about the intervention, as well as about the exercises that compose the intervention.

Item 4 – Description and consideration of the context’s characteristics in intervention modeling: described in item 2–12 (setting in which the exercises are performed).

## Second stage: feasibility and piloting

Item 5 – Description of the pilot test and its impact on the definitive intervention.

The pilot intervention aimed to determine the feasibility, acceptability, and practicality of the postpartum exercise program. The pilot intervention is described elsewhere [58].

After the pilot intervention, participants were asked to provide feedback on satisfaction with training sessions, type of exercises, and perceived improvement in physical fitness, using a Likert scale (1 – not satisfied at all / do not agree at all; 2 – not satisfied / do not agree; 3 – indifferent; 4 – satisfied / agree; 5 – very satisfied / strongly agree) for questions 1 to 3, and 1 – yes; 2 – no; 3 – same, for the other questions, as follows:

1. 90.9% of participants reported being very satisfied with the program, while 9.1% reported being satisfied.
2. 100% reported being very satisfied with the exercise professional.
3. 45.5% agreed that it is more motivating in a small group setting than one-on-one, while 45.5% strongly agreed. 9.1% did not agree.
4. 100% reported an improvement in their fitness levels, namely in terms of strength (81.8%), cardiorespiratory fitness (54.6%), flexibility (36.4%), posture (81.8%), body composition (72.7%) and balance and coordination (45.5%).
5. 90.9% of women reported increased levels of physical activity, while 9.1% reported that they were equally active.
6. 100% reported feeling more energy for daily activities and less stress.
7. All participants reported that they would recommend the training program and confirmed their participation in it in a possible future postpartum period.

There were no clear differences in opinion/satisfaction between online and in-person participants. The final version of the postpartum exercise program was adjusted in accordance with the opinions of the participants of the pilot intervention [46].

## Third stage: evaluation

Item 6 – Description of the control condition (comparator) and reasons for the selection.

Recent systematic reviews have been showing the effectiveness of physical activity and exercise-based interventions during the postpartum period on several maternal outcomes such as weight loss [59], postpartum fatigue [20], depressive symptoms [16, 17, 59–61], urinary and fecal incontinence [19], and lumbo-pelvic pain [62]. Very low-quality evidence recommends specific exercise programs in the treatment of diastasis recti postpartum [63], and no systematic reviews addressing functional and fitness parameters were found.

However, it is unclear which features are included in the most effective exercise programs regarding outcomes and long-term adherence [28]. There is also a great variability across trials in the components of exercise programs, modes of delivery, follow up times and outcome measures [60].

Saligheh et al. [59] showed that the studies identified as most likely to associate with postpartum depression and/or weight loss changes were those with supervision (either 1–1 or group), structure (weekly frequency, scheduled durations and moderate intensity), which adhered to specific exercise guidelines over an extended postpartum period (e.g., 12 weeks+), and were supplemented by several psycho-social support strategies (e.g., educational information and exercise advice).

Therefore, the control conditions of the present structured and supervised postpartum exercise program were the following: health, level of physical activity, quality of life, low back pain and pelvic pain, fatigue, depression, and functional and fitness level (Table 3).

**Table 3:** Control conditions of the structured and supervised postpartum exercise program

Pre-exercise assessment (early and later postpartum)	Postural and functional assessment (early and later postpartum)	Fitness assessment (early and later postpartum)	Fitness assessment (later postpartum)
Health: PAR-Q+ [29] Level of Physical Activity: IPAQ [31] Quality of life: WHOQOL-Bref [33] Pelvic pain: PGQ [35] Low back pain: RMDQ [37] Fatigue: FAS [39] Depression: EPDS [41]	Postural assessment: Static observation of the anatomical references, in the frontal and sagittal planes, verifying the symmetry in relation to the imaginary midline [44] Functional assessment: DNS: The seated diaphragm test, Intra-abdominal pressure test, Quadruped rockforward test [51] FMS: Shoulder Mobility, Active straight leg raise, Deep Squat, Rotary Stability [52, 53]	Blood pressure, resting heart rate and reserve and maximum heart rate calculated [23] Body composition: BMI; Waist-hip ratio; Electrical Bioimpedance [23]	Cardiorespiratory Fitness: Rockport One-Mile Fitness Walking Test [54, 55] Muscular resistance: maximum number of arm extensions (push-ups) [23]; “Chair Stand Test” [56] Flexibility: V sit and reach test [23, 57]

The pilot study showed that the intervention with a tailored and supervised exercise program for the postpartum period, with a minimum duration of 12 weeks, performed three times a week, produced positive effects on health and fitness parameters [58]. However, a study protocol of a larger controlled trial should be developed in order to verify the impact of this program on the control conditions under study (i.e., level of physical activity, quality of life, low back and pelvic pain scales, level of fatigue, level of depression, functional and fitness levels).

Item 7 – Description of the strategy for delivering the intervention within the study context.

The exercise program was planned to be delivered in spaces certified for the practice of physical exercise (i.e., gyms and health clubs), or in healthcare facilities (e.g., clinics, hospitals, health units), as well as online (i.e., at home or outdoor), as long as it is supervised by graduated and qualified postpartum exercise professionals with the skills described in the “Pregnancy and Postpartum Exercise Specialist” educational standards by EuropeActive [64].

Item 8 – Description of all materials or tools used in the delivery of the intervention: this content was addressed in item 2: sub-items 2.1 and 2.12.

Item 9 – Description of fidelity of the delivery process compared the study protocol.

The aim of the present study was to build and preliminary validate a postpartum exercise program. This was organized into three distinct periods, with specific exercises for each stage, considering the pre-defined objectives, as well as the principle of individuality. The exercise program aimed to be safe and effective in relation to selected parameters of health and physical fitness, as described in Table 3.

Item 10 – Description of a process evaluation and its underlying theoretical basis.

The evaluation process was designed to determine the effectiveness of the exercise program. Participant assessments were performed prior to the exercise intervention (preferably between four and eight weeks postpartum), during the exercise intervention (after eight weeks), and at the end of the intervention (after another eight weeks). In case the participant started the intervention later (between eight and 16 weeks) she would be evaluated before and after 12 weeks of exercise intervention. The effectiveness of the exercise program was analyzed through the results found in these different evaluation moments, using the instruments described in Table 3 (item 6).

Item 11 – Description of internal facilitators and barriers potentially influencing the delivery of the intervention as revealed by the process evaluation.

Facilitators potentially influencing the delivery of the intervention were related to the availability of facilities and equipment necessary to deliver the exercise program, as well as the ability to carry out the intervention in person or remotely. The fact that the mothers could take the babies to the sessions, the training schedule and their environment were also facilitators. The expertise of the exercise professionals and the educational materials and communication were also facilitators.

Potential barriers were identified as the lack of support from health professionals and the family, the fatigue, the lack of motivation and confidence, the time restrictions due to the care of the newborn. Another of the concerns presented by women at this period of life is whether breastfeeding is impaired by the practice of physical exercise.

These aspects were taken into account when producing educational materials for free distribution, such as the guides “Active Pregnancy Guide - Physical activity, nutrition, and sleep” [47, 48], and “Promotion of physical activity and exercise during pregnancy and postpartum. Health professionals guide” [49, 50], and by free access videos posted on the YouTube channel “Gravidez Ativa – Active Pregnancy”^1^.

Item 12 – Description of external conditions or factors occurring during the study which might have influenced the delivery of the intervention or mode of action.

External conditions or factors occurring during the study which might influence the delivery of the intervention were related to the fact that the recruitment and referral processes also depend on the advice of healthcare professionals.

Item 13 – Description of costs or required resources for the delivery of the intervention.

The exercise program was designed to be delivered in proper training spaces. These facilities and sports equipment must follow all the required safety and hygiene standards, which implies costs. These costs were also needed to support human resources, as the exercise program was planned and structured to be delivered by qualified exercise professionals, with knowledge in sports and exercise sciences. There were also costs with research and evaluation equipment used. Thus, the implementation of the exercise program required specific costs such as facilities, equipment, dissemination, and qualified professionals.

## Discussion

Current evidence supports the importance of developing specific exercise programs for postpartum recovery with the goal of returning to pre-pregnancy condition. However, the literature search revealed that postpartum exercise-based interventions are lacking. Moreover, interventions in physical activity and exercise lack homogeneous methods of development, implementation and evaluation due to their complexity, and there are no structured models of intervention in exercise specifically for the postpartum period.

Therefore, it becomes extremely important to develop and validate reproducible and effective physical exercise programs that promote health and physical fitness during the postpartum period. A physical exercise program can be considered a complex intervention, since it is adapted to a specific population and environment and is affected by several components regarding efficacy and safety. Thus, the need arises to develop and validate well-defined and replicable exercise protocols to fill the identified gaps. The CReDECI2 [12] has the potential to help professionals in the development and planning of complex interventions, such as an exercise program, filling these gaps. A pilot study was developed to evaluate the feasibility of the postpartum exercise program and its potential impact on maternal health and fitness parameters [58].

The main limitation of the study is that, although the three stages of CReDECI 2 [12] were followed, this process does not guarantee the effectiveness of the intervention. Another limitation is that the pilot intervention included a small number of participants (less than 20), which may have limited the number and diversity of feedback received. Nevertheless, participants’ opinions were sufficiently homogeneous and positive to conclude that the intervention was properly prepared and implemented. Furthermore, this process does not guarantee the absence of obstacles in the design, implementation, or evaluation of a future larger-scale study. Therefore, a study protocol of a larger controlled trial should be developed in order to verify the impact of this program on the control conditions under study (i.e., level of physical activity, quality of life, low back and pelvic pain scales, level of fatigue, level of depression, functional and fitness levels). Moreover, future developments regarding the supporting educational materials foresee a website and an informatics application (“app”) to provide guidance to women and professionals.

The CReDECI2 is a formally consensed reporting guideline aiming to improve the reporting quality of the development and evaluation stages of complex interventions in healthcare. This tool has been used previously in the development of complex interventions that included physical exercise tailored to different populations such as cancer survivors [65], frail older adults [66] and pregnant women [67]. So far, this is the first study applying the CReDECI2 guideline to a population of postpartum women. Regarding the present study, the aim of using this guideline was to comprehensively report the development of a tailored postpartum exercise program, considered as a complex intervention since it contains several interacting components, to enhance transparency and prepare a study protocol for the future development of a multicenter RCT. The present work presented a more complete description for research and practice, and provided guidance to develop a study protocol with a larger sample in order to prove the effectiveness of a supervised postpartum exercise program on selected parameters of health. Future multicenter study should include more participants in diverse in-person and online contexts with similar lengths of participation and follow-up. The main goal of future research is to improve the guidelines for postpartum exercise, to be useful to assist health and exercise professionals and researchers in the planning, promotion and implementation of complex interventions and trials, and to incorporate this program into a routine healthcare setting.

## Conclusion

The CReDECI2 process has the potential to help practitioners to develop, plan and report on complex interventions, such as an exercise program, as well as translating research into clinical practice. The CERT provided a reproducible framework for clear description of the exercise program. A postpartum exercise program and supporting materials were developed and preliminary validated by exercise professionals and the target population. The exercise program includes several components that can be adjusted to the context and characteristics of postpartum women. The present work provided guidance to develop a study protocol with a larger sample in order to prove the effectiveness of a supervised postpartum exercise program on selected parameters of health and fitness.

## Data Availability

The datasets generated and/or analysed during the current study are available in the IPSANTAREM repository: https://repositorio.ipsantarem.pt/. The materials (videos and ebooks) are available for free.

## Footnotes

1 YouTube channel. Gravidez Ativa – Active Pregnancy: https://www.youtube.com/channel/UC0Vyookwc0mcQ5T70imtoNA/playlists Accessed September 1, 2023.
